# 672. Improving Splint Compliance in Burn ICU Patients Through the Use of Rehabilitation Boards

**DOI:** 10.1093/jbcr/irag033.553

**Published:** 2026-04-06

**Authors:** Vitina Kammin, Midori Stewart, Viviana Rubio, Maria Tavarez

**Affiliations:** HCA Florida Kendall Hospital, Miami, FL; HCA Florida Kendall Hospital, Miami, FL; HCA Florida Kendall Hospital, Miami, FL

## Abstract

**Introduction:**

Burn injuries present complex rehabilitation challenges that extend beyond the acute phase. Early and consisting splinting is vital in burn rehabilitation, yet compliance in the ICU is often inconsistent. Multiple factors contribute to this challenge, including variability in order clarity, difficulty coordinating care among staff, patient discomfort, and lack of understanding of splinting schedules. To address this, rehabilitation splint boards were implemented in May 2025 in the Burn Intensive Care Unit (BICU) at an ABA-verified center through a multidisciplinary nursing and rehabilitation therapy initiative.

**Methods:**

Rehabilitation splinting boards were placed on the wall in front of each patient bed (12 rooms), displaying the most frequently used splints as well as the splint schedules and positioning. Education was delivered to BICU nursing staff, patient care techs (PCTs), and the inpatient therapists. Compliance was measured through chart review and observation. ICU staff and rehabilitation therapists completed a survey on ease of use, clarity, and impact on workflow.

**Results:**

Post implementation (4 months review), compliance with splinting orders and splint utilization improved by over 75%; adherence to board usage was at 96%. Staff completing survey reported clearer orders, easier implementation, and better team communication. The boards enhanced collaboration between nursing and rehabilitation therapy while reducing ambiguity and improving accountability.

**Conclusions:**

Rehabilitation boards proved to be a simple, low-cost intervention that significantly improved splint compliance and staff satisfaction in the Burn ICU. This multidisciplinary quality improvement initiative standardized communication and practice, supporting optimal patient outcomes. Expansion to additional units and evaluation of long-term recovery impact with regards to scar contracture management are warranted.

**Applicability of Research to Practice:**

Rehabilitation boards improve splint compliance by clarifying orders and enhancing collaboration between nursing and rehabilitation therapy. This low-cost, visual intervention translates evidence-based burn rehabilitation practices into routine ICU care, supporting standardized care and better patient outcomes.

**Funding for the study:**

N/A.

